# Is COVID-19 an Independent Risk Factor for Heparin-Induced Thrombocytopenia?

**DOI:** 10.7759/cureus.13425

**Published:** 2021-02-18

**Authors:** Samragnyi Madala, Michael Krzyzak, Shabnam Dehghani

**Affiliations:** 1 Medicine, Staten Island University Hospital, Staten Island, USA; 2 Medicine, Touro College of Osteopathic Medicine, New York, USA

**Keywords:** covid-19, heparin induced thrombocytopenia, mural arch thrombus, unfractionated heparin, low molecular weight heparin

## Abstract

Severe acute respiratory syndrome coronavirus 2 (SARS-CoV-2) causes a viral respiratory illness initially described in Wuhan, China, and was declared a pandemic by World Health Organization (WHO) in 2020, and the disease is named coronavirus disease (COVID-19). SARS-CoV2 is known to cause fever, cough, fatigue, and acute respiratory distress syndrome. As more patients become infected, extrapulmonary manifestations came to rise and hypercoagulability is one among those. COVID-19 could predispose patients to both venous and arterial thromboembolic events which are commonly treated with unfractionated heparin or low molecular weight heparin (LMWH). The treatment of patients who develop heparin-induced thrombocytopenia (HIT) while being treated with heparin or LMWH for COVID-induced thromboembolic complications is challenging.

We describe a patient admitted to the hospital with COVID-19 pneumonia, found to have a cerebrovascular event treated with unfractionated heparin. She also received therapeutic LMWH for anticoagulation on day 1 of presentation due to atrial fibrillation. She was diagnosed with HIT and was found to have a pulmonary embolism, aortic arch mural thrombus, and arterial thrombi in the lower extremities. As more recent studies showed HIT antibodies in COVID-19 patients who are naive for heparin-based products, COVID-19 may be an independent risk factor for the development of HIT. The role of COVID-19 in the development of HIT is uncertain. High vigilance is required to diagnose and initiate treatment for HIT early in the disease course as it can be life-threatening.

## Introduction

In December 2019, cases of pneumonia of unknown etiology were first reported in Wuhan, Hubei province in China. The etiology was found to be viral in nature and called severe acute respiratory syndrome coronavirus 2 (SARS-CoV-2) [1]. The disease was named coronavirus disease (COVID-19) by World Health Organization (WHO), which was later declared as a global pandemic on March 11, 2020 [2]. As of January 2021, there are 95 million confirmed cases of SARS-CoV-2 with two million deaths worldwide [3]. Along with respiratory symptoms, complications related to hypercoagulability are predominant in these patients. Our case describes a patient who initially presented with a cerebrovascular event likely related to COVID-19. Treatment with unfractionated heparin caused a life-threatening adverse reaction called heparin-induced thrombocytopenia (HIT).

## Case presentation

A 65-year-old female with a history of chronic obstructive pulmonary disease, and hypothyroidism presented to the emergency department (ED) with shortness of breath which was progressively worsening two days prior to presentation. She also reported having a fever with a temperature of 38 degrees Celsius ( ͦC), chills, and nonproductive cough in the last two days. The patient denied exposure to sick contacts, recent travel outside the state, or recent immobilization. Her temperature on admission was 39 ͦC, blood pressure 132/80 mmHg, heart rate 110 beats per minute, respiratory rate of 18 per minute. Physical examination was significant for lethargy, bilateral rales on auscultation of lung fields. Her oxygen saturation was 88% while on 5 liters per minute delivered through nasal cannula so she was placed on noninvasive positive pressure ventilation. Chest X-ray showed bilateral interstitial opacities consistent with viral pneumonia. The patient tested positive for SARS-CoV-2. Laboratory results at the time of admission are presented in Table 1.

**Table 1:** Labs on admission. WBC count: white blood cell; PLT: platelet count; BMP: basic metabolic profile; BUN: blood urea nitrogen; CRP: C-reactive protein; PT: prothrombin time; PTT: partial thromboplastin time; INR: international normalized ratio.

	Patient data	Normal range
Hematology		
WBC	15.32 K/uL	3.8-10.5 K/uL
Hemoglobin	13.2 g/dL	11.5-15.5 g/dL
Hematocrit	43.7%	34.5-45.0%
PLT	313 K/uL	150-400 K/uL
BMP		
Sodium	138 mmol/L	135-145 mmol/L
Potassium	4.9 mmol/L	3.5-5.3 mmol/L
BUN	17.0 mg/dL	7.023 mg/dL
Creatinine	1.3 mg/dL	0.5-1.3 mg/dL
CRP	2.82 mg/dL	0.00-0.40 mg/dL
Ferritin	101 ng/mL	15-150 ng/mL
Coagulation profile		
PT	15.50 sec	9.8-13.1 sec
PTT	29.7 sec	27.5-36.3 sec
INR	1.35	0.88-1.17
D-dimer	1079 ng/ml	0-230 ng/mL

**Figure 1:**
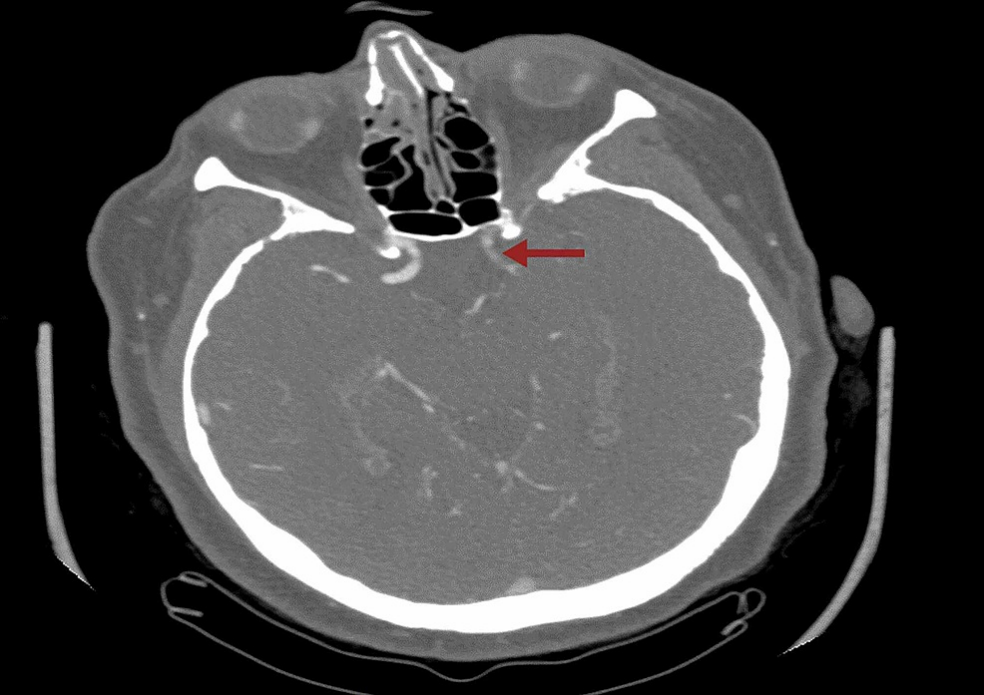
CT perfusion study showing occlusion of the supraclinoid segment of the left ICA. ICA: internal carotid artery.

She was started on remdesevir and dexamethasone as per institutional guidelines for the treatment of COVID-19 viral pneumonia. A 12-lead electrocardiogram revealed atrial fibrillation with a ventricular rate of 120 per minute, so anticoagulation was started with low molecular weight heparin (LMWH) 1mg/kg dosing twice daily and rate-controlling medications were administered. 12 hours after admission to the hospital, right arm motor drift, facial droop, and dysarthria were noted. National Institute of Health Stroke Scale (NIHSS) was 3. Emergent CT of the head revealed age indeterminate lacunar infarct in the left basal ganglia, with no intracranial hemorrhage. CT perfusion study of head and neck showed occlusion of the supraclinoid segment of the left internal carotid artery (ICA) with occlusive thrombus extending into the proximal left anterior cerebral artery (ACA) and middle cerebral artery (MCA) (Figure 1).

Left ACA opacified via an anterior communicating artery. Corresponding large 126 ccs of ischemic penumbra involving the left frontal, temporal and parietal lobes, basal ganglia were also seen. Anticoagulation was switched from LMWH to unfractionated heparin (UFH) in anticipation of the revascularization procedure. The case was discussed with an Interventional neurologist and the decision was made not to proceed with revascularization due to low NIHSS. While the patient was being monitored in the stroke unit, on day 3 of admission, she developed new right-sided hemiplegia, aphasia with altered mentation. The emergent CT of the head showed an increasing size of the infarct within the left basal ganglia and corona radiata. CT perfusion showed stable left ICA/MCA occlusion, however, due to progressive symptoms she underwent emergent left ICA/MCA thrombectomy. The weakness improved after revascularization with an increase in muscle strength from 1/5 to 3/5 in both right upper and lower extremities. UFH was discontinued due to an elevated risk of bleeding post revascularization.

**Figure 2:**
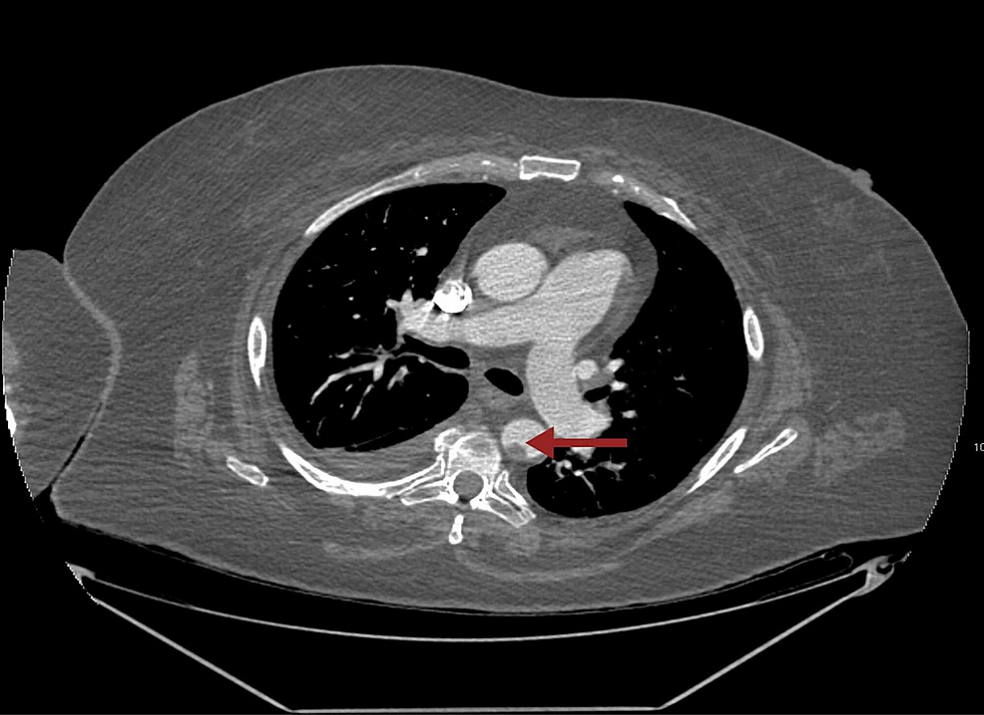
CT angiogram of the chest with the arrow pointing towards mural thrombus within the descending thoracic aorta.

On day 7 after the procedure, anticoagulation was restarted with LMWH due to coexisting atrial fibrillation and COVID-19 infection. At this point, the patient completed a 10-day course of remdesevir and dexamethasone and was saturating well with 3 liters per minute supplemental oxygen through the nasal cannula. two days after restarting anticoagulation, a stroke code was called due to sudden onset, left lower extremity weakness. CT perfusion of head and neck demonstrated new distal left M2 branch occlusion within the Sylvian fissure. Mural thrombi were noted in the distal aortic arch and descending aorta. A complete angiogram of the chest and abdomen was done to determine the extent of clot burden. CT angiogram of the chest confirmed the presence of mural thrombus (Figure 2).

**Figure 3:**
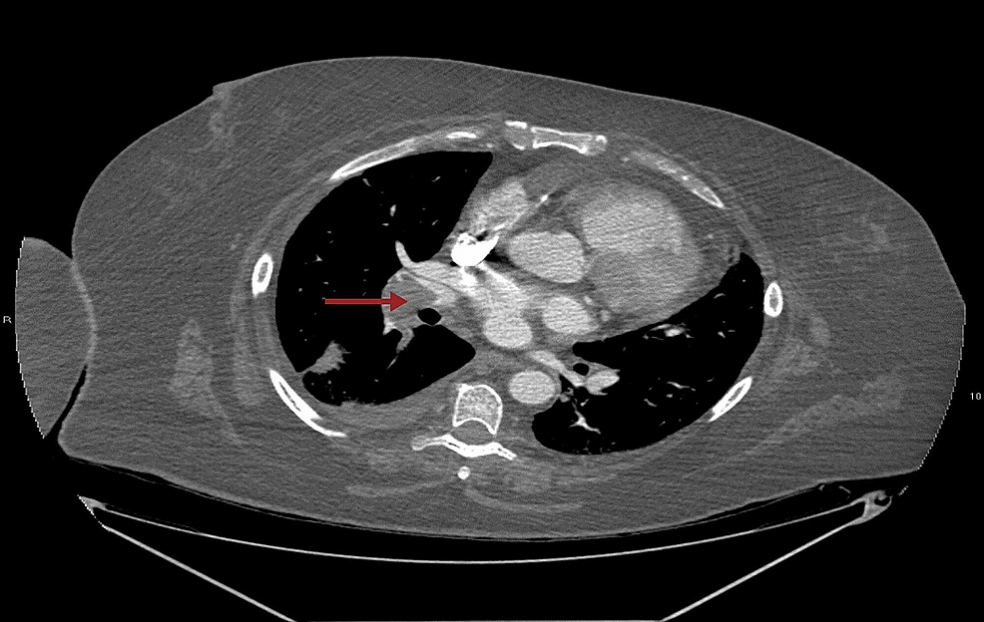
CT angiogram of the chest with the arrow pointing at a large pulmonary embolus within the right interlobar pulmonary artery.

**Figure 4:**
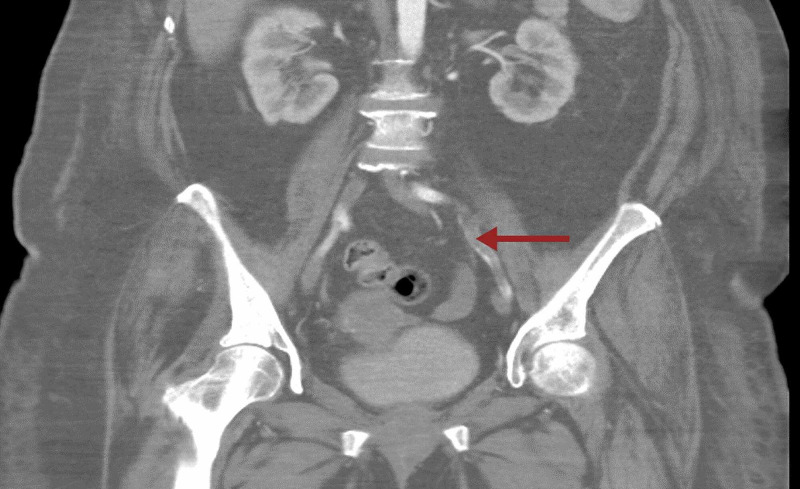
CT angiogram of the abdomen showing long segment thrombus in the external iliac artery.

**Figure 5:**
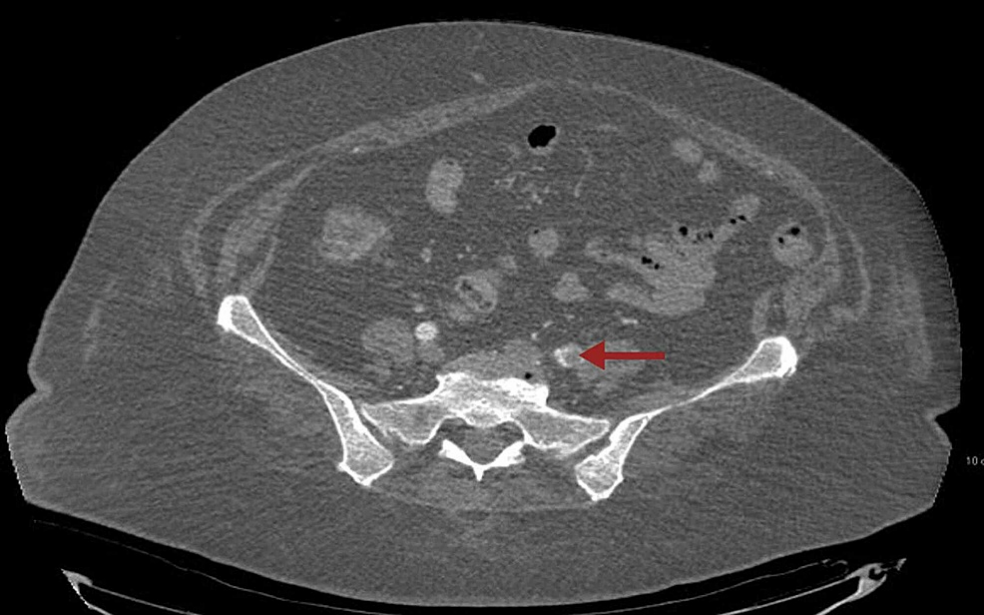
CT angiogram of the abdomen with the arrow pointing towards thrombus in the left common femoral artery.

**Figure 6:**
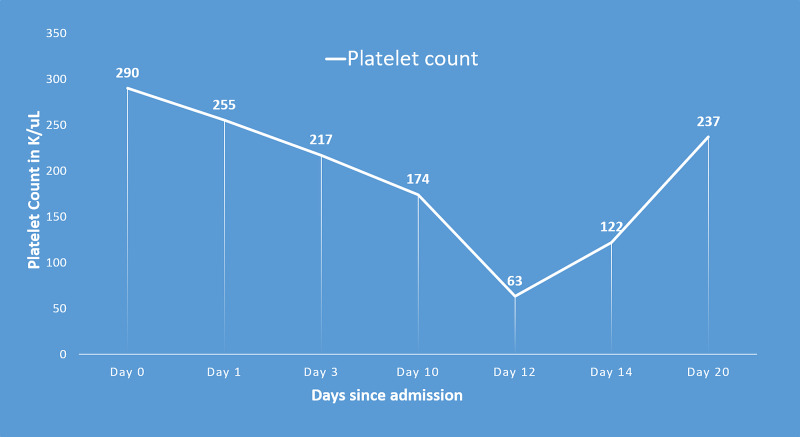
Trend in platelet count over course of admission demonstrating drop by more than 50% as HIT developed. Timeline of events with relation to heparin administration and platelet count: (1) Day 0: First day of LMWH administration; (2) Day 1: Unfractionated heparin administration; (3) Day 3: Unfractionated heparin discontinued; (4) Day 10: Restarted LMWH; (5) Day 12: The first day that the platelet count fall exceeds 50%; new-onset thrombosis detected; LMWH discontinued. anti-PF4/heparin antibodies and SRA sent; (6) Day 14: Antibodies returned positive. LWMH: low molecular weight heparin; HIT: heparin-induced thrombocytopenia; PF4: platelet factor 4; SRA: serotonin release assay test.

Along with that, a large filling defect was noted within the right interlobar pulmonary artery with extension into the right middle and lower lobe pulmonary artery as well as segmental and subsegmental branches of the right lower lobe compatible with pulmonary embolism (Figure 3). CT angiogram of the abdomen showed thrombus in left common femoral artery extending into proximal internal iliac as well as left external iliac arteries (Figures 4, 5). There is a reconstitution of flow within both arteries distally. Platelets dropped from 174 to 63 within few days after restarting anticoagulation (Figure 6). 4T score for screening of HIT was 6 (2 each for greater than 50% drop and nadir >30, timing within 5-10 days of exposure and new thrombosis). LMWH was switched to argatroban infusion. Platelet factor 4 antibody test and serotonin release assay test (SRA) were positive (Table 2).

 

 

 

**Table 2:** Labs pertinent to HIT diagnosis. HIT: heparin-induced thrombocytopenia; PTT: partial thromboplastin time; PF4: platelet factor 4.

Labs pertinent to HIT diagnosis	
Platelet count	63 K/μL (normal range: 150-400 K/μL)
PTT	31.8 sec (normal range: 27.5-36.3 sec)
D-dimer	5315 ng/mL (normal range: 0-230 ng/mL)
Heparin-PF4 antibody	9.7 μ/mL (normal range: 0.0-0.9μ/mL)
Serotonin releasing assay	94% (normal: negative)

The patient underwent a left lower extremity angiogram by vascular surgery and treated with tissue plasminogen activator catheter-directed thrombolysis (tPA-CDT). Revascularization was successful with three-vessel runoff and dopplerable pulses in the left lower extremity. Anticoagulation was subsequently switched to apixaban. The patient recovered from COVID-19 and was discharged to the nursing home for rehabilitation for stroke.

## Discussion

SARS-CoV-2 infected patients present mainly with upper respiratory symptoms such as nonproductive cough, fever, myalgias, headache, and sore throat. Whereas most patients recover after having mild symptoms, some progress into severe pneumonia, septic shock, acute respiratory distress syndrome (ARDS), and multiorgan failure. Laboratory studies show normal or decreased lymphocyte count (60%) and elevated C-reactive protein due to active inflammation [4]. Initial presentation primarily focuses on respiratory findings on early CT scans showing multiple patchy ground-glass opacities, predominantly in the peripheral zone of the lungs [4].

As the virus infects more people, newer complications, mainly related to hypercoagulability are being noticed. In a study done in New York City on inpatient intensive care unit (ICU) and non-ICU patients on venous thromboembolic (VTE) prophylaxis, the percentage of thrombotic events were 29.4% and 11.5%, respectively, and included both venous and arterial thrombi [5]. SARS-CoV-2 is a single-stranded RNA virus that infects the human host through angiotensin-converting enzyme 2 (ACE2) receptor which is expressed in the lung, kidney, intestines, heart, and even endothelial cells [6]. Zsuzsanna et al demonstrated viral elements, along with inflammatory cells within the endothelial cells leading to endotheliitis [6]. The elevated risk of thrombosis was likely due to endotheliitis in microcirculation leading to hypercoagulability from platelet activation [5]. Thromboembolic events ranged from a pulmonary embolism, cerebrovascular events, cardiac thrombi, and lower limb arterial and venous occlusions. platelet count, D-dimer, prothrombin time, and troponin are some of the indicators for thrombotic disease [7]. Li et al demonstrated that older patients diagnosed with SARS-CoV-2 and the presence of risk factors such as hypertension, diabetes, and history of cerebrovascular disease are at risk of developing cerebrovascular events [8]. Our patient initially presented with a cerebrovascular event requiring treatment with unfractionated heparin and later had a complicated course with HIT.

HIT is a prothrombotic adverse drug reaction to heparin derivatives like UFH or less commonly LMWH. HIT develops due to the formation of platelet-activating IgG antibodies against platelet factor 4 (PF4)/heparin complex. HIT is an immune-mediated, life-threatening condition, with the development of unexpected thrombocytopenia and new arterial or venous thrombosis about a week after exposure to heparin derivatives [9]. 4T score is a commonly used clinical scoring system to assess the likelihood of HIT. It includes the degree of thrombocytopenia, timing of the drop in platelet count, associated thrombosis, and presence of other causes of thrombocytopenia with 2 points given to each criterion [10]. In those with moderate to high probability of HIT, diagnosis can be confirmed with HIT IgG-specific antibodies (anti-PF4/H) and SRA. The risk of developing thrombosis is at least 50% in serologically proven cases of HIT which is 12 times higher than matched controls [9]. When the probability of HIT is high, the recommendation is to stop heparin and switch to a non-heparin-based anticoagulant. Parenteral direct thrombin inhibitors like argatroban, fondaparinux, and direct oral anticoagulants, factor Xa inhibitors such as rivaroxaban, dabigatran, and apixaban; are commonly used for the treatment of HIT [11].

As previously mentioned, the virus enters the cell through ACE2 receptors and completes replication. An immune response is then triggered in the form of a cytokine storm in infected epithelial and endothelial cells. Cytokine storm is characterized by the tremendous release of proinflammatory cytokines like tumor necrosis factor-α, interleukin-6 (IL-6), interleukin-1β. Of these, IL-6 is the main mediator of cytokine storm in COVID-19 patients [12]. Earlier studies demonstrated a higher predominance of anti-PF4/H antibodies in intense proinflammatory conditions associated with increased levels of IL-6 [13]. Furthermore, there might be a higher risk of HIT antibody formation in COVID-19 patients than other medically ill patients due to high IL-6. It is noteworthy in a recent study performed in Boston to detect anti-PF4/H antibodies in admitted COVID-19 patients who received more than five days of UFH, 12% had positive HIT antibodies at 25 days [14]. Liu et al demonstrated HIT antibodies among ICU patients in heparin naïve and patients treated with LMWH. This also raises the question of COVID-19-related “spontaneous” HIT syndrome [15,16]. Spontaneous HIT syndrome has been described as an autoimmune prothrombotic thrombocytopenic disorder in patients with no heparin exposure. It was predominantly reported in inflammatory states like orthopedic surgeries and infections raising the possibility of its association with COVID-19 [17].

## Conclusions

COVID-19 could be an independent risk factor for the development of HIT, though the mechanism is unclear. In addition, due to the high risk of thromboembolic events in COVID-19, anticoagulation with heparin is increasingly being used in hospitalized patients. It is very important to have a high suspicion for HIT in these critically ill patients as it can be life-threatening and early detection can lead to an improvement in patient outcomes.
